# Effect of Serum Spermidine on the Prognosis in Patients with Acute Myocardial Infarction: A Cohort Study

**DOI:** 10.3390/nu14071394

**Published:** 2022-03-27

**Authors:** Zhecong Yu, Yundi Jiao, Jin Zhang, Qianyi Xu, Jiahui Xu, Ruixue Li, Wei Yuan, Hui Guo, Zhaoqing Sun, Liqiang Zheng

**Affiliations:** 1 Department of Cardiology, Shengjing Hospital of China Medical University, Shenyang 110004, China; zhecongyu@126.com (Z.Y.); jiaoyundi1987@163.com (Y.J.); xqy306672924@126.com (Q.X.); xujiahui1116@163.com (J.X.); 2 School of Public Health, Shanghai Jiao Tong University School of Medicine, Shanghai 200025, China; 3 Department of Laboratory Medicine, Shengjing Hospital of China Medical University, Shenyang 110004, China; zjin052412@163.com; 4 School of Public Health, China Medical University, Shenyang 110122, China; liruixue2020@163.com (R.L.); yuanweilnyk@163.com (W.Y.); huiguo0471@163.com (H.G.)

**Keywords:** major adverse cardiac events, spermidine, oxidative stress, prognosis, mediation analysis

## Abstract

Background: Spermidine, a natural polyamine, was found critically involved in cardioprotection and lifespan extension from both animal experiments and human studies. Aims: This study aimed to evaluate the effect of serum spermidine levels on the prognosis in patients with acute myocardial infarction (AMI) and investigate the potential mediation effect of oxidative stress in the above relationship. Methods: We included 377 patients with AMI in a prospective cohort study and measured serum spermidine and oxidative stress indexes (superoxide dismutase enzymes, glutathione peroxidase, and Malondialdehyde) using high-performance liquid chromatography with fluorescence detector and enzyme-linked immunosorbent assay, respectively. The associations of spermidine with AMI outcomes were evaluated using Cox proportional hazards models. Results: 84 (22.3%) major adverse cardiac events (MACE) were documented during a mean follow-up of 12.3 ± 4.2 months. After multivariable adjustment, participants with serum spermidine levels of ≥15.38 ng/mL (T3) and 7.59–5.38 ng/mL (T2) had hazard ratio (HR) for recurrent AMI of 0.450 [95% confidence interval (CI): 0.213–0.984] and 0.441 (95% CI: 0.215–0.907) compared with the ≤7.59 ng/mL (T1), respectively. Participants in T3 and T2 had HR for MACE of 0.566 (95% CI: 0.329–0.947) and 0.516 (95% CI: 0.298–0.893) compared with T1. A faint J-shaped association was observed between serum spermidine levels and the risk of MACE (*p*-nonlinearity = 0.036). Comparisons of areas under receiver operator characteristics curves confirmed that a model including serum spermidine levels had greater predictive power than the one without it (0.733 versus 0.701, *p* = 0.041). A marginal statistically significant mediation effect of superoxide dismutase was shown on the association between spermidine and MACE (*p* = 0.091). Conclusions: Serum spermidine was associated with an improved prognosis in individuals with AMI, whereas the underlying mechanism mediated by oxidative stress was not found.

## 1. Introduction

Cardiovascular diseases (CVDs) seriously threaten the quality of life, and its lifetime risk is over 30% [1]. Acute myocardial infarction (AMI) is the major cause of CVD death [2], and its recommended management is focusing on the improvement of prognosis [3]. Utilizing biomarkers in clinical practice could guide prognostic stratification of individuals with AMI [4]. A perfect biomarker might help delve into the pathophysiology of, find new targets for treatment of, and develop primary and secondary prevention measures of CVDs. Unfortunately, available biomarkers for AMI prognosis have not yet been fully explored.

Spermidine is a naturally occurring polyamine found in abundance in certain foods, such as wheat germ, natto, soybeans, aged cheese, and mushrooms [5]. Spermidine feeding in mice with heart failure (HF) reduced cardiovascular pathologies, including hypertension and cardiac dysfunction [6]. High levels of dietary spermidine intake were linked to lower blood pressure (BP) and decreased incidence and mortality of CVDs in humans [6,7,8]. Currently, the main methods for measuring serum spermidine concentration were by its content in the daily food intake, which may not reflect the accurate level of spermidine concentration in the human body due to self-reporting bias [7]. The systemic levels of spermidine are dependent on dietary intake, synthesized by intestinal microbiota and cellular metabolism [5], and thus it may be more reasonable to measure the spermidine concentration in human biological samples. Spermidine levels in the right atrial appendage were detected in individuals with congestive HF by high-performance liquid chromatography. Left ventricular ejection fraction and heart rate were found positively related to spermidine concentration [9]. The potential mechanisms that link spermidine to cardiovascular protective effects are not fully understood. Oxidative stress defense plays a vital role in the above relationship [5,6]. The harm of oxidative stress on the cardiovascular system is well recognized [10,11]. Spermidine could induce the production of antioxidant factor—Hemeoxygenase-1 [12]—decrease the cardiac mitochondrial reactive oxygen species levels [13], and protect against oxidative stress by autophagy [14,15]. However, to the best of our knowledge, findings were limited to animal or in vitro experiments, little is known from epidemiological studies.

Therefore, this study aimed to investigate the prognostic implication of serum spermidine in individuals with AMI, and to explore whether it impacts prognosis by regulating the underlying mechanisms by the regulation of oxidative stress levels.

## 2. Methods

### 2.1. Study Population

In total, 404 patients with AMI were admitted to Shengjing Hospital of China Medical University between May 2019 and January 2020. They were diagnosed using a cardiac troponin I level exceeding the 99th percentile of a normal reference population and with at least one of the following: chest pain lasting >20 min, diagnostic serial electrocardiographic changes consisting of new pathologic Q waves, or ST-segment and T-wave changes [16]. Participants with known malignancy or severe hepatic and renal insufficiency were excluded from the study. Sociodemographic, clinical, and biochemical data were available from the medical records. All the patients received standard medical treatment at the discretion of the attending physician, including emergency percutaneous coronary intervention (PCI), monitoring of the electrocardiogram, BP and blood oxygen saturation, dual antiplatelet therapy, and lipid-lowering therapy. In addition, beta-blockers, angiotensin-converting enzyme inhibit/angiotensin receptor blocker therapy should be given if BP and heart rate are tolerated.

The biological samples and clinical data of patients were obtained by the way of de-labeling personal information of the participants in order for full protection of privacy in this study. Additionally, institutional review board (IRB) approval of this study was exempted by Shengjing Hospital of China Medical University Research Ethics Committee, since our biological samples were collected from the remaining serum after routine clinical diagnosis and treatment.

### 2.2. Measurement of Serum SPERMIDINE and Oxidative Stress

The blood sample was obtained within 24 h of admission. Serum was stored at −80 °C in the Biobank of Shengjing Hospital of China Medical University after secondary centrifugation for 10 min at 3000 rpm, until assayed in a single batch for blinded determination of serum spermidine and oxidative stress levels. Briefly, the main process for serum spermidine concentration detection was as follows: First, spermidine trihydrochloride was performed by adding 200 μL 0.1 M HCl to 100 μL serum. Then, adding 1 ml acetonitrile (ACN) to the mixture to precipitate the serum protein. Finally, extracted samples were analyzed using high-performance liquid chromatography with fluorescence detector (HPLC-FLD) after derivatization. The mobile phase solution A and B were ultra-pure water and ACN, respectively. Gradient elution was selected at 0–7 min, 55–50% A; 7–25 min, 50–10% A; 25–31 min, 10% A; 31–35 min 10–55% A; 35–40 min 55% A, while other detection conditions were kept unchanged. The oxidative stress levels of participants were evaluated by measuring specific antioxidant factors—superoxide dismutase (SOD) enzymes, glutathione peroxidase (GPX)—and the index of lipid peroxidation—Malondialdehyde (MDA) [11,17]. The serum oxidative stress levels were detected by enzyme-linked immunosorbent assay (ELISA), including the double antibody sandwich method for SOD (Cloud-Clone, Wuhan, China) and GPX (Jonln, Shanghai, China) and the competitive inhibition enzyme-linked immunosorbent assay for MDA (Cloud-Clone, Wuhan, China). The microwell plate was used as the solid phase, and the detection was manually performed.

### 2.3. Follow-Up and Endpoints

Of the 404 recruited participants, 5 died during hospitalization and 32 provided incorrect contact information, and the remaining 377 were included in the final analysis. A telephone follow-up was conducted between June 2020 and March 2021. The investigators first explained the purpose of the follow-up to patients and obtained their informed consent. The investigators were blinded to patients’ spermidine through a standardized questionnaire to obtain detailed information of clinical endpoints. The questionnaire included demographic information, records of outcomes, such as time of onset and the address of the hospital, and drug use. The clinical endpoint was the report of major adverse cardiovascular events (MACE), including recurrent AMI, strokes, HF, CVD deaths, and all-cause mortality. Death information was informed by the participants’ immediate family members.

### 2.4. Statistical Analysis

Baseline continuous variables are presented as mean with standard deviation and compared with analysis of variance (ANOVA), while categorical variables are shown as numbers with percentages and compared with chi-square test or the Fisher exact test. Participants were grouped into tertiles according to the spermidine levels: T3, ≥15.38 ng/mL; T2, 7.59–15.38 ng/mL; T1, ≤7.59 ng/mL. Risk of incident MACE was determined by Cox proportional hazards model. Hazard ratio (HR) and 95% confidence interval (CI) were calculated. The covariates included in the Cox regression models were depended on the results of univariate analysis (*p* < 0.20) and the documented traditional risk factors. We constructed two models: an unadjusted model 1 and a fully adjusted model 2 with covariates of age, sex, new AMI, smoking status, serum triglycerides, cardiac troponins I levels, ST-segment elevation myocardial infarction (STEMI) or non-STEMI, Killip II/III class, PCI, and medication intake status including aspirin, statin, clopidogrel, beta-blocker, ticagrelor, and angiotensin-converting enzyme inhibit/angiotensin receptor blocker. In the above models, spermidine was entered as a categorical variable with T1 as the reference group. We also used restricted cubic splines with three knots at the 5th, 50th, and 95th centiles to flexibly model the association between serum spermidine levels and risk of MACE with adjusted model 2. To assess the impact on the model’s predictive value of adding in risk factors, the change in −2 log-likelihood was calculated for each model (model 1 and model 2) and compared with a χ^2^ distribution. In addition, we calculated the prognosis index of the Cox model with/without spermidine levels, constructed receiver operating characteristic (ROC), and computed the areas under the curves (AUC) and Youden’s index to assess the predictive power of each Cox model. The associations between SOD, GPX, and MDA (as categorical variables by tertiles) and the risk of MACE were calculated by Cox proportional hazards regression analysis with adjusted model 2. The associations between SOD, GPX, MDA, and spermidine were computed by multiple linear regression. Mediation analysis was performed to examine the indirect effect of oxidative stress levels on the association between spermidine and MACE. Serum levels of spermidine, SOD, GPX, and MDA, and incidence of MACE risk were log10 transformed and then standardized with z-score standardized prior to the mediation analyses.

The primary analysis was performed with IBM SPSS 26.0 (SPSS Inc., Chicago, IL, USA). Cox model with a restricted cubic spline was conducted using SAS software (version 9.3; SAS Institute Inc., Cary, NC, USA). The ROC analysis was performed by MedCalc 18.2.1 (MedCalc Software Ltd., Ostend, Belgium) and the mediation analysis was performed by AMOS 23.0 (IBM, New York, NY, USA). A two-tailed *p* value less than 0.05 was accepted as indicating statistical significance.

## 3. Results

### 3.1. Patient Characteristics

Demographic and clinical characteristics of the participants were demonstrated in Table 1. The study included 275 males (72.9%) and 102 females (27.1%) with a median age of 53.0 (62.0, 71.0) years. In total, 187 (49.6%) were diagnosed with STEMI and 314 (83.3%) with new AMI. The median serum spermidine level was 11.0 (6.0, 17.7) ng/mL. AMI cases were divided into tertiles according to the spermidine levels. Participants with high spermidine levels were more likely to be older and have a lower triglycerides level; participants with intermediate spermidine levels were less likely to be smokers. There was no significant difference for baseline sex, new AMI, stroke, hypertension, diabetes, systolic and diastolic BP, heart rate, creatinine, total cholesterol, low-density lipoprotein, high-density lipoprotein, fasting glucose, cardiac Troponins I, STEMI, Killip II/III class, PCI, medical treatment aspirin, statin, clopidogrel, beta-blocker, ticagrelor, angiotensin-converting enzyme inhibit/angiotensin receptor blocker, SOD, GPX, and MDA among T1, T2, and T3. (all *p* ≥ 0.05).

### 3.2. Endpoints

There were 47 (12.5%) recurrent AMI, 10 (2.7%) strokes, 4 (1.1%) HF, 16 (4.2%) CVD deaths, and 25 (6.6%) all-cause mortality during a mean follow-up time of 12.3 ± 4.2 months. In total, there were 84 (22.3%) MACE: 37 in T1 (≤7.59 ng/mL) group, 23 in T2 (7.59–15.38 ng/mL) group, and 24 in T3 (≥15.38 ng/mL) group were observed. Univariate Cox regression analysis showed that age, sex, smoking, Killip II/III class, aspirin, statin, beta-blocker, and ticagrelor were predictors of MACE (Table 2). Multivariate Cox regression analysis revealed that (Table 3) in model 1, patients with spermidine ≥15.38 ng/mL (T3) versus spermidine ≤7.59 ng/mL (T1) had a lower risk of recurrent AMI [unadjusted hazard ratio (HR): 0.479; 95% confidence interval (CI): 0.233–0.982]. No significant differences were observed between spermidine and the risk of MACE (T3 versus T1: unadjusted HR, 0.656; 95% CI, 0.392–1.096 and T2 versus T1: unadjusted HR, 0.620; 95% CI, 0.368–1.043). There were also no differences in strokes, HF (data not shown), CVD deaths, or all-cause mortality between different spermidine level groups. In the fully adjusted model (model 2), spermidine remained a significant predictor of recurrent AMI (T3 versusT1: adjusted HR, 0.450; 95% CI, 0.213–0.948 and T2 versus T1: adjusted HR, 0.441; 95% CI, 0.215–0.907). Overall, 43.4% and 48.4% risk reduction rates of MACE were shown in T3 and T2 group, respectively, compared with that in the T1 group (adjusted HR: 0.566; 95% CI: 0.329–0.974 and 0.516; 0.298–0.893, respectively). There remained no differences in the risk of strokes, HF, CVD deaths, or all-cause mortality between different spermidine level groups.

### 3.3. Restricted Cubic Splines

**Figure 1:**
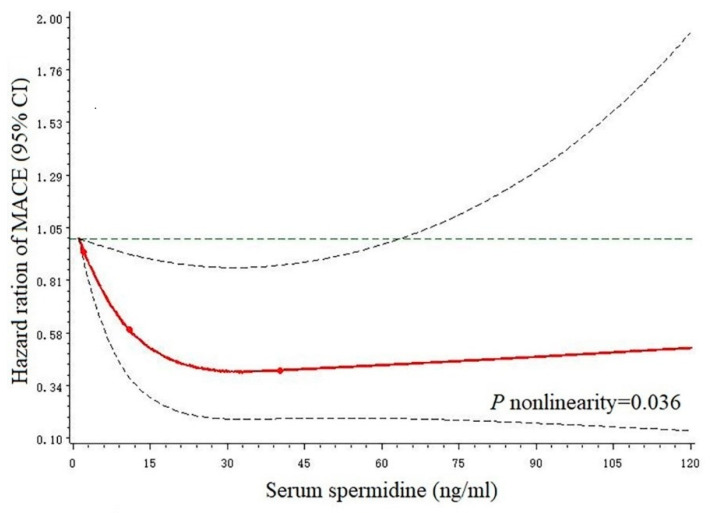
Relationship of serum spermidine with MACE in patients with AMI (*n* = 377). Hazard ratios and 95% CIs were derived from restricted cubic spline regression, with knots placed at the 5th, 50th, and 95th percentiles of the distribution of serum spermidine levels. Red lines indicate hazard ratios, the green line indicates the reference line (The minimum of spermidine as a reference point), and dashed lines indicate 95% CI. Hazard ratios were adjusted for the same variables as model 2 in Table 3. MACE major adverse cardiac events, CI confidence interval, AMI acute myocardial infarction.

In Figure 1, we used restricted cubic splines to flexibly model and visualize the relationship between predicted spermidine and MACE. The risk of MACE decreased rapidly afterward until around 30 ng/mL of predicted spermidine levels and then started to relatively flat (*p*-nonlinearity = 0.036). A faint J-shaped association between spermidine levels and the risk of MACE was observed.

### 3.4. ROC Curves

**Figure 2:**
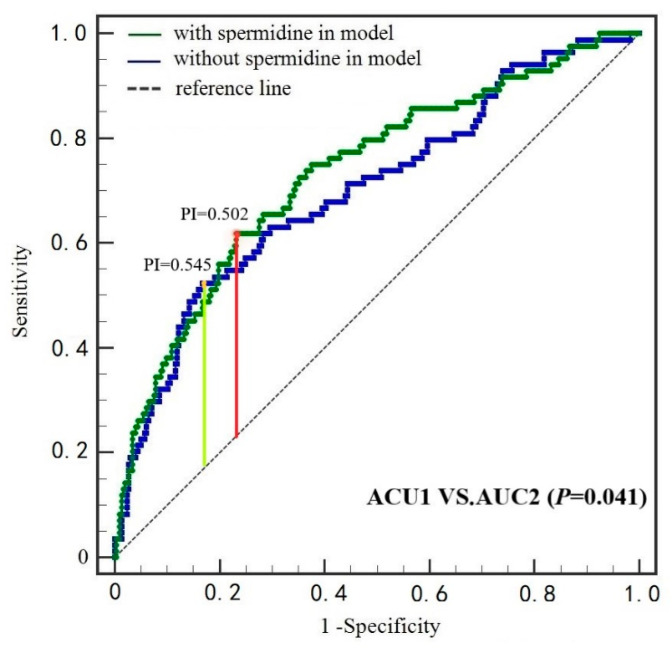
Receiving operating characteristics curves for MACE from Cox proportional hazards models with and without spermidine in patients with AMI (*n* = 377). In the model with spermidine, the optimal cut-off value for PI was 0.502 at Youden’s index of 0.387, shown in red. In the model without spermidine, the optimal cut-off value for PI was 0.545 at Youden’s index of 0.357, shown in yellow. Comparison between the AUCs is noted in the bottom panel. MACE: major adverse cardiac events, AMI: acute myocardial infarction, PI: prognosis index, AUC: areas under the curves.

The ROC curves for the risk of MACE from Cox models without/with spermidine were shown in Figure 2. With MACE as the outcomes, the first model included age, sex, new AMI, smoking, triglycerides, cardiac troponins I, STEMI, Killip II/III class, PCI, aspirin, statin, clopidogrel, beta-blocker, ticagrelor, and angiotensin-converting enzyme inhibit/angiotensin receptor blocker had an AUC of 0.701 (95% CI: 0.652–0.747, *p* < 0.001), a change in −2 log-likelihood of 37.597. The second model included spermidine levels and saw a significant increase (*p* = 0.009) in the model fit [change in −2 log-likelihood of 6.825 with 1 df, AUC was 0.733 (95% CI: 0.685–0.777, *p* < 0.001)].

### 3.5. Mediation Analysis

**Figure 3:**
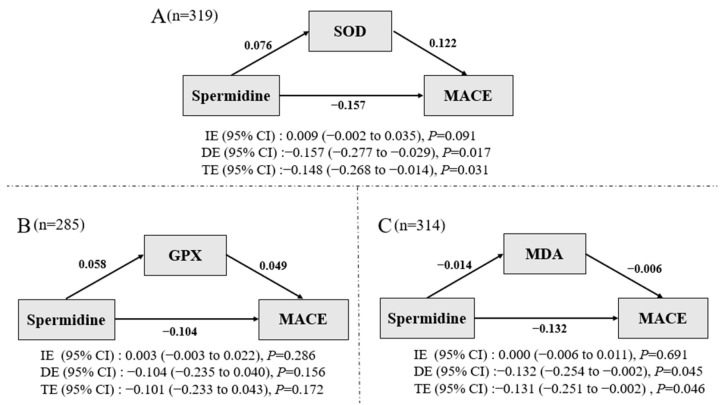
(**A**–**C**) Mediation effect of SOD (*n* = 319), and GPX (*n* = 285), MDA (*n* = 314) on the MACE association. CI: confidence interval, SOD: superoxide dismutase, MDA: Malondialdehyde, GPX: glutathione peroxidase, IE: indirect effect, DE: direct effect, TE: total effect. Mediation effect with 95% CI was noted in the panel.

We did not find any significant associations between SOD, GPX, MDA, and MACE. Non-significant relationships were found between SOD, GPX, MDA, and spermidine levels (all *p* ≥ 0.05, data not shown). Figure 3A–C demonstrated the direct, indirect, and total effect of spermidine levels on MACE risk through SOD, GPX, and MDA, respectively. The standardized negative direct effect of spermidine though SOD and MDA on MACE was [−0.157 (−0.277–−0.029), *p* = 0.017] and −0.132 (−0.254–−0.002), *p* = 0.045], respectively. No statistical significance was found in the standardized negative direct effect of spermidine on MACE though GPX [−0.104 (−0.235–0.040), *p* = 0.156]. There was a marginal statistical significance in standardized positive indirect effect through SDO for spermidine on MACE [0.009 (−0.002–0.035), *p* = 0.091]. Non-significant indirect effect through GPX or MDA was detected. The standardized negative total effect of spermidine on MACE though SOD, GPX, and MDA was [−0.148 (−0.268–−0.014), *p* = 0.031], [−0.101 (−0.233–0.043), *p* = 0.172], and [−0.131 (−0.251–−0.002), *p* = 0.046], respectively.

## 4. Discussion

In this prospective cohort study, we explored the effect of spermidine on the prognosis in patients with AMI and measured serum spermidine concentration using HPLC-FLD assay. Our findings suggested that higher serum spermidine levels were associated with decreased risk of recurrent AMI and MACE. Serum spermidine levels had a faint J-shaped association with the risk of MACE. Besides, compared with a model incorporating conventional risk factors alone, adding spermidine in the model significantly improved its predictive ability (*p* = 0.041). Moreover, we found a marginal statistically significant effect of spermidine on MACE through SOD (*p* = 0.091).

### 4.1. Spermidine and MACE

As a natural polyamine, Spermidine could be supplemented by daily food intake to increase intracellular concentration and exert its physiological function [5,18]. As a result, in animal experiments, spermidine supplementation in water reversed age-related arteriosclerosis. These improvements were related to the reduction in structural factors that induce stiffness in the arterial wall [15]. Consistently, spermidine improved left ventricular diastolic function, prevented cardiac hypertrophy, and reduced diastolic function from developing, which delayed progressions of HF and myocardium [6]. This study indicated that participants with high serum spermidine levels reported a lower risk of recurrent AMI after adjustment of conventional risk factors. Discrepancies in evidence between animal experiments and human studies should be addressed because of the species differences. In fact, due to ornithine decarboxylase antizyme maintaining the concentration of spermidine in cells [19], the physiological function of spermidine concentration in intracellular homeostasis needs a prospective trial with repeated measurements to explore. It was interesting to note that dietary intake of polyamines might be associated with an increased risk of colorectal adenoma in females [20]. Inversely, this team found that high polyamine intake was negatively associated with the risk of colorectal cancer in females with body mass index ≤ 25 kg/m^2^ in a subsequent cohort study [21]. Thus, an interventional trial was performed to evaluate whether regular intake of polyamines or spermidine provides substantial protection against CVD and tumor growth to prolong human life. To our knowledge, this study was the first one to demonstrate an inverse relationship between serum spermidine levels and the risk of MACE after AMI. The association emerged as independent of other risk factors. Spermidine and spermine were enriched in the whole blood of centenarians [22]. Longitudinal and nutritional studies might be helpful to find potential therapeutic. Increased polyamines levels were associated with hemodynamic overload. Spermidine levels were positively related to left ventricular ejection fraction and heart rate [9]. Notably, these cross-sectional studies with small sample size, to some extent, suggested that polyamine levels might be associated with CVDs risks and longevity. Findings in our study indicated that AMI patients with a higher level of spermidine might have a better prognosis. Furthermore, prospective cohort studies have consistently found that dietary intake of spermidine could reduce the risk of cardiovascular disease and death, characterized by a decrease in soluble N-terminal B-type natriuretic peptide (NT-proBNP, a key clinical biomarker of HF and BP) [6,7,8]. Similar protective effect of spermidine was also verified in our prospective cohort of 377 patients with AMI. Interestingly, our study was the first one that detected a faint J-shaped association between spermidine levels and the risk of MACE. In animals, intracerebroventricular or intracarotid injections of spermidine and spermine were found to disrupt the blood–brain barrier integrity within 15 min and were associated with the formation of vasogenic brain oedema [23]. Nutrient or adequate spermidine intake may be recommended in humans. In a word, findings in our study that serum spermidine levels were independently associated with the risk of recurrent AMI and MACE were not only a supplement to the previous studies, but also provided evidence for future clinical trials in verifying the application of spermidine in CVD prevention. Further, a randomized placebo-controlled trial found that oral lactis and arginine, a precursor of spermidine, improved endothelial function in healthy individuals. This may reduce the risk of atherosclerosis [24].

### 4.2. Potential Mechanistic Links

The protective effects of spermidine in cardioprotection were closely related to autophagy [5]. Spermidine increased the levels of autophagy markers and reverses arterial aging [15], improved AMI-induced cardiac dysfunction by promoting AMPK/mTOR-mediated (enzymes involved in the regulation of autophagy) autophagic flux [25], and promoted protective autophagy and mitophagy in cardiomyocytes [6]. Suppressing oxidative stress was an essential link in the physiological function of spermidine through autophagy. Animal experiments revealed that spermidine could reduce the damage from oxidative stress by inhibiting inflammation and accumulation of free radicals in cells [6,15]. Spermidine suppressed oxidative stress damage and inflammatory cytokines in rats post-myocardial infarction, characterized by the increase in the levels of SOD and the decrease in the levels of MDA in cardiomyocytes [25]. Spermidine could reduce pro-oxidants levels such as reactive oxygen species and increase antioxidants levels such as SOD [26]. In this study, to our knowledge, we are the first try to find this potential mechanism wherein spermidine exerted cardioprotection by alleviating oxidative stress in humans. Regrettably, we did not find any significant associations among SOD, GPX, MDA, spermidine, and MACE risk. A marginal statistically significant effect of spermidine on MACE through SOD (*p* = 0.091) might indicate the underpower of this study. Moreover, the differences in the number and categories of metabolite transporters, and the discrepancies in physiological concentrations of spermidine in cells between humans and animals should be addressed.

### 4.3. Merits and Limitations

The main strength of this study was that we measured serum spermidine levels using the HPLC-FLD assay, which was more objective and accurate than food frequency questionnaire, and the latter may introduce self-reporting bias. Additionally, we also measured the serum oxidative stress indexes (SOD, GPX, and MDA) to explore the potential mechanism underlying the protective effect of spermidine on CVDs in humans, findings from this study might guide the clinician in prognostic stratification of AMI patients to prevent a new major cardiovascular event. Some limitations should be considered in the light of the present results. Firstly, we only recruited the study patients with AMI, and additional patients with coronary heart disease were not included; In addition, the present study individuals were recruited from the admitted AMI patients in a single hospital and the outpatients of multi-center settings were not recruited, which induced some selection bias and limited the generalizability of the present results. Secondly, spermidine was measured at a single time point and we did not collect more time-point blood samples during follow-up, especially at the end of the observation. Dynamic changes of spermidine levels after discharge may provide additional predictive information concerning the effect of spermidine on the prognosis in patients with AMI. Thirdly, in the present study, we did not collect some potential confounders such as the contents and frequency of food intake and history of medications use (antibiotics and herbal medicine et al.), which may induce some confounding bias. Finally, the study may have been underpowered, considering the broad range of the 95% CI and the mechanism mediated by SOD reached a marginal statistical significance (*p* = 0.091).

## 5. Conclusions

Serum spermidine might be a potential biomarker that predicted AMI prognosis. Higher serum spermidine levels were associated with lower risk of recurrent AMI and MACE. Spermidine could additionally improve the predictive power of the conventional risk factors for MACE. We did not find a significant effect of spermidine in cardioprotection by regulating the levels of oxidative stress in humans.

## Figures and Tables

**Table 1:** Demographics for patients admitted to hospital with AMI stratified by spermidine tertiles.

Characteristics	Combined	T1 (≤7.59 ng/mL)	T2 (7.59~15.38 ng/mL)	T3 (≥15.38 ng/mL)	*p*-Value
Ages, years	53.0 (62.0, 71.0)	59 (51.0, 67.0)	62.0 (54.0, 73.3)	65.0 (55.8, 72.3)	0.003
Females, %	102 (27.1)	28 (22.4)	44 (34.9)	30 (23.8)	0.050
New AMI, %	314 (83.3)	103 (82.4)	104 (82.5)	107 (84.9)	0.834
Previous histories					
Stroke, %	46 (12.2)	13 (10.4)	12 (9.5)	21 (16.7)	0.168
Hypertension, %	220 (58.4)	64 (51.2)	74 (58.7)	82 (65.1)	0.083
Diabetes, %	95 (25.2)	29 (23.2)	33 (26.2)	33 (26.2)	0.820
Smoking, %	227 (60.2)	86 (68.8)	67 (53.2)	74 (58.7)	0.037
Systolic BP, mmHg	130.0 (116.0, 145.5)	129.0 (116.0, 143.5)	129.5 (118.0, 144.3)	130.5 (115.0, 150.0)	0.771
Diastolic BP, mmHg	79.3 ± 14.2	79.7 ± 14.2	78.2 ± 14.0	80.1 ± 14.5	0.799
Heart rate, beats/min	77.0 (67.0, 87.0)	78.0 (68.0, 88.0)	76.0 (66.0, 86.0)	76.0 (65.0, 88.0)	0.782
Creatinine, µmol/L	70.6 (60.7, 84.9)	70.0 (61.7, 87.1)	69.0 (59.0, 83.0)	73.2 (62.3, 86.3)	0.354
Total cholesterol, mmol/L	4.6 (3.8, 5.2)	4.60 (3.9, 5.3)	4.6 (3.8, 5.1)	4.3 (3.7, 5.2)	0.648
Low-density lipoprotein, mmol/L	0.9 (0.8, 1.1)	0.9 (0.8, 1.2)	0.9 (0.8, 1.1)	0.9 (0.8, 1.1)	0.720
High-density lipoprotein, mmol/L	2.8 (2.2, 3.5)	2.7 (2.2, 3.3)	2.9 (2.1, 3.5)	2.8 (2.2, 3.5)	0.758
Triglycerides, mmol/L	1.5 (1.0, 2.4)	1.5 (1.1, 3.0)	1.5 (1.0, 2.2)	1.4 (0.9, 2.1)	0.040
Fasting glucose, mmol/L	6.1 (5.2, 7.8)	6.1 (5.3, 7.8)	6.0 (5.2, 7.5)	6.2 (5.2, 8.5)	0.881
Cardiac Troponins I, μg/L	9.2 (1.28, 34.9)	9.7 (1.3, 35.5)	6.4 (1.0, 27.6)	11.6 (1.4, 37.0)	0.284
ST-segment elevation, %	187 (49.6)	71 (56.8)	54 (42.9)	62 (49.2)	0.087
Killip II/III class, %	38 (10.1)	11 (8.8)	11 (8.7)	16 (12.7)	0.489
PCI, %	273 (72.4)	94 (75.2)	96 (76.2)	83 (65.9)	0.130
Medical treatment					
Aspirin, %	364 (96.6)	120 (96.0)	121 (96.0)	123 (97.6)	0.723
Statin, %	363 (96.3)	118 (94.4)	122 (96.8)	123 (97.6)	0.373
Clopidogrel, %	261 (69.2)	94 (75.2)	82 (65.1)	85 (67.5)	0.192
Beta-blocker, %	245 (65.0)	78 (62.4)	83 (65.9)	84 (66.7)	0.753
Ticagrelor, %	112 (29.7)	28 (22.4)	43 (34.1)	41 (32.5)	0.088
ACEI/ARB, %	228 (60.5)	71 (56.8)	70 (55.6)	87 (69.0)	0.054
SOD ^a^, ng/mL	53.7 (43.5, 66.0)	53.8 (40.3, 65.1)	53.7 (45.0, 66.8)	53.6 (43.6, 65.8)	0.630
GPX ^b^, ng/mL	78.3 (33.9, 154.6)	70.9 (30.9, 139.5)	88.6 (39.5, 170.9)	69.7 (35.6, 194.4)	0.433
MDA ^c^, μg/mL	16.2 (10.4, 20.1)	15.4 (9.1, 18.6)	16.3 (11.1, 20.2)	16.6 (11.0, 21.0)	0.188

Data were presented as mean ± SD, numbers (percentages), or median (interquartile range). ^a^ included 319 patients for analysis, ^b^ included 285 patients for analysis, ^c^ included 314 patients for analysis. AMI: acute myocardial infarction, BP: blood pressure, PCI: percutaneous coronary intervention, ACEI: angiotensin-converting enzyme inhibit, ARB: angiotensin receptor blocker, SOD: superoxide dismutase, MDA: Malondialdehyde, GPX: glutathione peroxidase.

**Table 2:** Univariable Cox regression analysis for MACE in Patients with AMI.

Characteristic	HR (95% CI)	*p*-Value
Ages, years	1.034 (1.016–1.051)	<0.001
Females, %	2.168 (1.405–3.346)	<0.001
New AMI, %	1.049 (0.600–1.837)	0.866
Previous histories		
Stroke, %	1.311 (0.712–2.417)	0.385
Hypertension, %	0.872 (0.568–1.341)	0.534
Diabetes, %	1.295 (0.811–2.068)	0.279
Smoking, %	0.535 (0.349–0.822)	0.004
Systolic BP, mmHg	1.001 (0.991–1.011)	0.888
Diastolic BP, mmHg	0.994 (0.979–1.010)	0.473
Heart rate, beats/min	1.008 (0.996–1.020)	0.210
Creatinine, µmol/L	1.000 (0.999–1.000)	0.718
Total cholesterol, mmol/L	0.993 (0.963–1.024)	0.649
Low-density lipoprotein, mmol/L	0.965 (0.781–1.193)	0.745
High-density lipoprotein, mmol/L	0.956 (0.800–1.142	0.618
Triglycerides, mmol/L	0.892 (0.768–1.035)	0.132
Fasting glucose, mmol/L	1.036 (0.966–1.111)	0.328
Cardiac Troponins I, μg/L	1.000 (0.999–1.001)	0.528
ST-segment elevation, %	1.176 (0.766–1.805)	0.460
Killip II/III class, %	2.313 (1.323–4.044)	0.003
PCI, %	0.831 (0.523–1.320)	0.432
Medical treatment		
Aspirin, %	0.408 (0.178–0.937)	0.034
Statin, %	0.309 (0.149–0.641)	0.002
Clopidogrel, %	1.241 (0.768–2.005)	0.377
Beta-blocker, %	0.591 (0.385–0.908)	0.016
Ticagrelor, %	0.572 (0.336–0.975)	0.040
ACEI/ARB, %	0.814 (0.529–1.251)	0.348

HR hazard ratio, CI confidence interval, MACE major adverse cardiac events, other abbreviations as in Table 1.

**Table 3:** Multivariable Cox regression analysis for recurrent AMI, strokes, CVD deaths, all-cause mortality, and MACE.

Outcomes	T1 (≤7.59 ng/mL)	T2 (7.59~15.38 ng/mL)	T3 (≥15.38 ng/mL)
No. of recurrent AMI	23	13	11
Incidence rate (per 1000 person months)	14.1	8.0	6.8
HR (95% CI)			
Model 1	Ref.	0.570 (0.289–1.125)	0.479 (0.233–0.982)
Model 2	Ref.	0.441 (0.215–0.907)	0.450 (0.213–0.948)
No. of strokes	5	2	3
Incidence rate (per 1000 person months)	2.9	1.2	1.8
HR (95% CI)			
Model 1	Ref.	0.416 (0.081–2.146)	0.652 (0.156–2.731)
Model 2	Ref.	0.184 (0.030–1.131)	0.293 (0.057–1.495)
No. of CVD deaths	6	5	5
Incidence rate (per 1000 person months)	3.5	2.9	3.0
HR (95% CI)			
Model 1	Ref.	0.839 (0.256–2.750)	0.853 (0.260–2.798)
Model 2	Ref.	0.845 (0.208–3.439)	0.616 (0.150–2.521)
No. of all-cause mortality	9	7	9
Incidence rate (per 1000 person months)	5.3	4.2	5.5
HR (95% CI)			
Model 1	Ref.	0.780 (0.290–2.094)	1.031 (0.409–2.599)
Model 2	Ref.	0.822 (0.271–2.498)	0.832 (0.285–2.427)
No. of MACE	37	23	24
Incidence rate (per 1000 person months)	23.9	14.8	15.6
HR (95% CI)			
Model 1	Ref.	0.620 (0.368–1.043)	0.656 (0.392–1.096)
Model 2	Ref.	0.516 (0.298–0.893)	0.566 (0.329–0.974)

Model 1 was unadjusted. Model 2 was adjusted for age, sex, new AMI, smoking, triglycerides, cardiac troponins I, STEMI, Killip II/III class, PCI, and treatment with aspirin, statin, clopidogrel, beta-blocker, ticagrelor, and angiotensin-converting enzyme inhibit/angiotensin receptor blocker. CVD: cardiovascular disease, MACE: major adverse cardiac events, HR: hazard ratio, CI: confidence interval. Other abbreviations as in Table 1.

## Data Availability

The data presented in this study are available on request from the corresponding authors. The data are not publicly available due to ethical, legal, and privacy issues.
